# The Hospital Funds and the Special Hospitals

**Published:** 1904-12-10

**Authors:** 


### THE HOSPITAL FUNDS AND THE SPECIAL HOSPITALS.

The *Medical Press* stated in its issue of the 30th ult. that the Sunday Fund urged in the most forcible way at their command—namely, by withholding a grant—the sale of the site of the Royal Orthopædic Hospital. Last week we pointed out that we could not credit this statement for the reasons given, and the following letter shows that there is not any foundation for the statement made by the *Medical Press* :—

THE CENTRAL FUNDS AND SPECIAL HOSPITALS.

I have just seen your article in last week's number dealing with comments made by the *Medical Press and Circular* with regard to the support given to special hospitals by King Edward's Hospital Fund for London and the disposal of the site in Oxford Street and Hanover Square, belonging to this hospital and formerly occupied by it.

With regard to the first matter I have nothing to do, but I was surprised to read that blame had been given to the Metropolitan Hospital Sunday Fund in the matter of the disposal of the site in question, and feel I cannot allow it to pass unnoticed.

At the time of writing I have not seen the article to which you draw attention, but desire to at once state that there is no truth whatever in the statement made by the *Medical Press and Circular* that the Metropolitan Hospital Sunday Fund took any part whatever in advising the hospital to dispose of its site or in offering any opinion as to its value.

I think it only fair to that Fund that this should be known, and I therefore will ask you to insert this letter in your next issue.

Tate S. Mansford,
Secretary of the Royal Orthopædic Hospital,
55 Bolsover Street, W.

December 5th, 1904.

The *Medical Press* has not yet produced " the host of small but deserving medical charities " from which grants have been withheld by the Sunday and King Edward's Hospital Funds. In its issue of the 7th inst. the *Medical Press* pleads " the pressure upon space and the verification of facts and figures, which may or may not be accepted as satisfactory reasons for the delay." The production of the list of " the host of small but deserving medical charities," which the writer of the article had before him, of course, when he wrote the original article, cannot have much relation to " the verification of facts and figures," and a month is surely long enough to enable the editor of our contemporary to find space for a list of this kind. It is promised, however, for next week ; and meanwhile we agree with our contemporary, in view of the above letter, that " the relations of the Royal Orthopædic Hospital to the Hospital Sunday Fund will require restating."

